# The Human Experience with Intravenous Levodopa

**DOI:** 10.3389/fphar.2015.00307

**Published:** 2016-01-06

**Authors:** Shan H. Siddiqi, Natalia K. Abraham, Christopher L. Geiger, Morvarid Karimi, Joel S. Perlmutter, Kevin J. Black

**Affiliations:** ^1^Department of Psychiatry, Washington University School of MedicineSt. Louis, MO, USA; ^2^School of Epidemiology, Public Health and Preventive Medicine, University of OttawaOttawa, ON, Canada; ^3^Department of Internal Medicine, University of WashingtonSeattle, WA, USA; ^4^Department of Neurology, Washington University School of MedicineSt. Louis, MO, USA; ^5^Programs in Occupational Therapy and Physical Therapy, Division of Biology and Biomedical Sciences, Departments of Neurology, Radiology, and Anatomy and Neurobiology, Washington University School of MedicineSt. Louis, MO, USA; ^6^Division of Biology and Biomedical Sciences, Departments of Psychiatry, Neurology, Radiology, and Anatomy and Neurobiology, Washington University School of MedicineSt. Louis, MO, USA

**Keywords:** levodopa, intravenous, Parkinson, DOPA, pharmacokinetics, carbidopa, FDA, IND

## Abstract

**Objective:** To compile a comprehensive summary of published human experience with levodopa given intravenously, with a focus on information required by regulatory agencies.

**Background:** While safe intravenous (IV) use of levodopa has been documented for over 50 years, regulatory supervision for pharmaceuticals given by a route other than that approved by the U.S. Food and Drug Administration (FDA) has become increasingly cautious. If delivering a drug by an alternate route raises the risk of adverse events, an investigational new drug (IND) application is required, including a comprehensive review of toxicity data.

**Methods:** Over 200 articles referring to IV levodopa were examined for details of administration, pharmacokinetics, benefit, and side effects.

**Results:** We identified 142 original reports describing IVLD use in humans, beginning with psychiatric research in 1959–1960 before the development of peripheral decarboxylase inhibitors. At least 2760 subjects have received IV levodopa, and reported outcomes include parkinsonian signs, sleep variables, hormone levels, hemodynamics, CSF amino acid composition, regional cerebral blood flow, cognition, perception and complex behavior. Mean pharmacokinetic variables were summarized for 49 healthy subjects and 190 with Parkinson's disease. Side effects were those expected from clinical experience with oral levodopa and dopamine agonists. No articles reported deaths or induction of psychosis.

**Conclusion:** At least 2760 patients have received IV levodopa with a safety profile comparable to that seen with oral administration.

## Introduction

Impairments in dopaminergic neurotransmission in the basal ganglia are a hallmark of Parkinson disease (PD), the second most common neurodegenerative disease. Replacement of dopamine has been the cornerstone of treatment for PD. Because dopamine itself does not cross the blood-brain barrier (BBB), its immediate precursor levodopa (l-3,4-dihydroxphenylalanine, l-DOPA) is administered since it crosses the BBB (Hornykiewicz, 1963; Cotzias et al., 1967; Birkmayer and Hornykiewicz, 2001). Although purified levodopa was first ingested by mouth in 1913 (Roe, 1997), it was first used for medical treatment by intravenous (IV) rather than oral administration (Pare and Sandler, 1959; Birkmayer and Hornykiewicz, 2001).

Oral levodopa has become the preferred method of treatment clinically, but IV levodopa administration still holds advantages over the oral form for some purposes. First, the rapid administration of IV levodopa is often necessary for certain study designs, including those focused on the pharmacokinetics and pharmacodynamics of the drug. Additionally, the IV route leads to more predictable plasma levodopa concentration because oral medications have highly variable absorption characteristics, especially in PD patients (Bushmann et al., 1989), with differences in absorption based on sex and age (Robertson et al., 1989; Kompoliti et al., 2002). IV administration also permits researchers to keep brain levodopa concentrations constant while assessing physiological responses over time. Recent years have seen increasing interest in potential benefits of continuous dopaminergic stimulation in the treatment of PD (Jenner et al., 2011). Continuous stimulation helps avoid wearing off of motor benefit during levodopa nadirs, and there is also some evidence that it may reduce the risk of, or mitigate, dyskinesias and other peak-dose side effects. Thus, IV levodopa may prove useful for human studies investigating the pathophysiology of continuous vs. pulsatile dopaminergic stimulation in humans. Finally, IV levodopa is sometimes used clinically in patients who cannot tolerate oral medications, such as PD patients during surgery or on total parenteral nutrition.

Current U.S. FDA regulations focus heightened scrutiny on research in which drugs are delivered by a route for which the drug has not been approved. Predictably, in addition to any safety benefits, the heightened scrutiny has created practical obstacles to research with IV levodopa, as described for instance by Rascol et al. (2001, p. 250). Specifically, an IND (Investigational New Drug) application must be submitted if the risks of IV administration significantly exceed those of oral levodopa [§21 CFR 312.2(b)(iii)]. Therefore, the overall goal of this paper is to determine whether or not IV levodopa carries risks greater than oral administration by compiling a literature review that comprehensively summarizes the human experience with intravenously administered levodopa. We tabulate the extent of human exposure, side effects, benefits, and efficacy. We also summarize pharmacokinetic (PK) and pharmacodynamic (PD) parameters from these studies. These data should help inform decisions about whether IV administration of levodopa requires an IND.

## Methods

The authors searched MEDLINE and OVID, reviewed selected books, searched toxicity databases, and followed references cited in those sources. Articles written completely in languages other than English, French, German, Italian, Spanish, or Portuguese were excluded. Search terms included (levodopa/L-dopa/DOPA) AND (intravenous/intravascular/infusion/injection/i.v.); limit to humans; search date through May, 2015. Studies using oral or intraduodenal l-DOPA administration were excluded except for PK/PD studies cited in **Table 2**. Studies in which IV levodopa was always coadministered with monoamine oxidase inhibitors (MAOIs) or catechol-O-methyltransferase (COMT) inhibitors were excluded. Levodopa methyl ester (Juncos et al., 1987) and d,l-DOPA (Pare and Sandler, 1959) were included, but PK/PD calculations were corrected for the difference in molecular weights. Co-administered drugs were reported if included by the authors.

We recorded total dose and maximum infusion rate. We also recorded pharmacokinetic (PK) and pharmacodynamic (PD) parameters where available, including steady state volume of distribution (VOD), clearance, distribution half life (*t*_½α_), elimination half life (*t*_½_ or *t*_β_), *E*_*max*_, and *EC*_50_. Reported data were used to calculate any missing PK parameters where possible. Additionally, any reports on efficacy were noted. Side effect frequency was recorded if reported. The number of subjects and subject conditions (Parkinson disease, other disease states or healthy volunteers) were recorded for each study. Average PK parameters were calculated across studies, weighted by the number of subjects.

## Results

One hundred forty-two articles reporting intravenous levodopa administration were identified. Most subjects with parkinsonism were diagnosed with idiopathic PD, but some studies reported a variety of etiologies including postencephalitic and vascular parkinsonism and PSP. PD patients differed in their history of prior drug treatment before the studies with conditions including *de novo*, fluctuating, on-off, and stable. Some subjects were treated with levodopa for conditions other than PD (see Table 1: Patient Populations and Response Parameters), including other movement disorders (dystonia, progressive supranuclear palsy [PSP], neuroleptic malignant syndrome [NMS], primary psychiatric disorders (schizophrenia, mood disorders, personality disorders), endocrine disorders (diabetes mellitus, essential obesity, hypopituitarism), hepatic disease (alcoholic cirrhosis, steatohepatitis, hepatic encephalopathy), cardiac valvular disease, and asthma. Healthy controls were also included in some studies.

**Table 1:** **Patient populations and response parameters**.

**Patient populations**	**Response parameters**
Healthy volunteers	Vital signs:
Movement disorders:	Heart rate, blood pressure,
Parkinson's (*de novo*, stable, fluctuators, on-off)	temperature, respirations
	Cardiovascular:
Progressive supranuclear palsy	ECG
Parkinson's disease psychosis	Cerebral blood flow
Carcinoma of the rectum	Renal:
Stereotactic surgery	Urine flow
Post-menopausal women	Urinary sodium excretion
Tourette syndrome/tic disorders	Potassium excretion
Asthma	Plasma renin activity
Schizophrenia	Renal plasma flow
Mood disorders:	Metabolism:
Mild to moderate depression	Urinary metabolite excretion
Treatment-resistant depression	Cerebral metabolism
Bipolar depression	Plasma metabolites
Cyclothymic disorder	CSF amino-acid composition
Borderline personality disorder	PD motor improvement
Neuroleptic malignant syndrome	Unified Parkinson's disease rating scale (UPDRS), walking, tapping, etc.
Hepatic disorders:	Dyskinesias
Alcoholic cirrhosis	Tic improvement
Steatohepatitis	Neuropsychiatric:
Hepatic encephalopathy	Cognition
Endocrine disorders:	Mood
Diabetes mellitus	Behavior
Essential obesity	Psychosis
Hypopituitarism	Dementia
Cardiovascular disease:	EEG (including REM sleep EEG)
Atrial septal defect	Endocrine:
Rheumatic valvular disease	Prolactin, HGH, ACTH, LH, vasopressin

*Subject populations given IV levodopa and responses to drug measured in studies listed in **Table 3***.

Pharmacokinetic data were reported for a total of 251 human subjects (see Table 2: Pharmacokinetics of Levodopa). Co-administration of a peripheral decarboxylase inhibitor (PDI) lowered the clearance and increased the elimination half-life of intravenously administered levodopa, while there was no notable effect of PDIs on volume of distribution. Additional PK data are available from studies that gave levodopa by other routes (Sasahara et al., 1980a; Poewe, 1993; Muhlack et al., 2004; LeWitt et al., 2009), and several studies report the bioavailability of oral doses relative to IV administration (Sasahara et al., 1980b; Robertson et al., 1989; Kompoliti et al., 2002).

**Table 2:** **Pharmacokinetics of levodopa**.

**References**	**Patient group**	**Clearance**		**Volume of distribution**		**Elimination half-life**		**Distribution half-life**		**Oral bioavailability**	
		***n***	**Mean (L/kg/h)**	***n***	**Mean (L/kg)**	***n***	**Mean (h)**	***n***	**Mean (h)**	***n***	**Mean (unitless)**
Birkmayer et al., 1973	PD	50	1.61	50	2.44	50	1.05				
Bredberg et al., 1990	Fluctuating	5	0.37								
Chan et al., 2004	*De novo*	12	0.36	12	0.63	12	2.25	12	0.17		
	Chronic	12	0.35	12	0.49	12	1.47	12	0.17		
Durso et al., 2000	“Slow” CD absorption					5	1.18				
	“Rapid” CD absorption					4	1.15				
Fabbrini et al., 1987	*De novo*	4	0.13	4	0.26	4	1.44				
	Stable	6	0.11	6	0.22	6	1.41				
	Wearing-Off	6	0.13	6	0.30	6	1.67				
	On-off	12	0.13	12	0.30	12	1.54				
Hardie et al., 1986^a^	Fluctuating	7	1.14	7	2.63	7	1.60	7	0.13		
Gancher et al., 1987^b^	*De novo*	5	0.34	5	0.56	5	1.70	5	0.10		
	Stable	4	0.33	4	0.62	4	1.80	4	0.11		
	Fluctuating	11	0.32	11	0.65	11	2.00	11	0.10		
Nutt et al., 1985	2 h IV	7	0.55	7	0.67	7	1.38	7	0.07		
(all PD, fluctuating)	2 h IV + PDI	7	0.30	7	0.80	7	2.01	7	0.11		
	≥20 h IV	4	0.52	4	0.88	4	1.19	4	0.11		
	≥20 h IV + PDI	4	0.28	4	1.09	4	2.60	4	0.33		
Nutt et al., 1992	*De novo*	8	0.44	8	0.75	8	1.60				
	Stable	12	0.42	12	0.75	12	1.70				
	Fluctuating	9	0.39	9	0.63	9	1.50				
Poewe, 1993^e^	PD		1.40		3.00		1.50		0.09		
Roberts et al., 1995^c^, ^d^	Healthy	8	0.37	8	1.13	8	2.15				
	Healthy + selegine	8	0.37	8	2.01	8	3.78				
Robertson et al., 1989^c^	Healthy elderly	9	0.85	9	1.01	9	0.82			9	0.63
	Healthy young	8	1.40	8	1.65	8	0.82			8	0.41
	Healthy elderly + PDI	8	0.35	8	0.62	8	1.23			8	0.85
	Healthy young + PDI	8	0.56	8	0.93	8	1.16			8	0.86
Sasahara et al., 1980b^f^	PD	5	1.38	5	1.29	5	0.65			5	0.33
Stocchi et al., 1992	Intravenous bolus	6	0.97	6	0.96	6	0.83				
(all “on-off”)	Intravenous infusions	2	0.63	2	0.82	2	0.90				
	Total *n*	212		242		251		73		16 (PDI) 22 (no PDI)	
	Weighted mean		0.719 L/kg/h		1.18 L/kg		1.50 h		0.14 h	0.86 (PDI) 0.48 (no PDI)	

*Summary of pharmacokinetic parameters with weighted means*.

a *Assumed mean weight to be 70 kg for VOD*.

b *Values read from graphs*.

c Half-life estimated from relationship: clearance = (ln 2 ^*^ VOD)/ elim. T1/2.

d *Assumed mean weight to be 70 kg for clearance*.

e *From a table with no additional data provided; not included in weighted mean calculations of pharmacokinetic parameters*.

f VOD estimated from relationship: clearance = (ln 2 ^*^ VOD)/ elim. T1/2.

The pharmacodynamic data (see Table 3: Reports of Human Experience with IV Levodopa) represent a total of 2760 human subjects, with a significant variety of patient groups and a multitude of response parameters (see Table 1). No side effects were reported for 1260 subjects. The highest total IV dose was 4320 mg in 1 day, given to a patient with idiopathic PD and carcinoma of the retina. The patient reported no adverse effects at this dose. The highest reported single bolus dose was 200 mg, and the highest infusion rate was 5.0 mg/kg/h.

**Table 3:** **The human experience with IV levodopa**.

**References**	***N***	**Diagnosis**	**PDI**	**Concomitant drugs**	**Total dose**	**Maximum rate**	**Side effects/comments**
Abramsky and Goldschmidt, 1974	4	Acute hepatic encephalopathy in cirrhotic patients with gastrointestinal bleeding	None mentioned	None mentioned	For several days (between 3 and 5 days depending on the patient)	600–1200 mg/day	Levodopa was administered intravenously with striking and rapid improvement of the comatose state. Within 2–5 h the patients had recovered their normal mental state
Aebert, 1967	11	10 PD, 1 post-encephalitis lethargica	None mentioned	None mentioned	75–1375 mg	75–100 mg/10–15 min	No side effects mentioned
Argyelan et al., 2008	15	PD	None mentioned	None mentioned	Not given	0.83 mg/kg/h	No side effects mentioned. Levodopa was associated with increases in learning-related activation in the left dorsal premotor cortex and in the right pre-supplementary motor area. In the former region, there was recovery of the normal activation response by levodopa. In the latter region, there was a treatment-mediated gain of response in that significant learning-related activation was present only when the patients were scanned on levodopa therapy
Baldy-Moulinier et al., 1977	19	Twelve alcoholic hepatic cirrhosis and hepatic encephalopathy; 3 alcoholic hepatic cirrhosis; 3 fatty liver (alcoholic) without cirrhosis; 1 healthy	None mentioned	None mentioned	125 mg	125 mg bolus	No effects on electroencephalogram, electrocardiogram, humeral arterial pressure, rectal temperature, cerebral perfusion or metabolism at this dose
Bara-Jimenez et al., 2003	15	Moderate to advanced PD	Carbidopa	KW-6002 (Adenosine A2A receptor antagonist)	Infusion of “optimal dose levodopa”	725 ± 65 ng/mL	No side effects mentioned for L-dopa plus placebo. There were no drug-related serious adverse events. Levodopa plus KW-6002 appeared generally safe and well-tolerated
Baronti et al., 1992	9	Moderate to severe PD (III–V)	Carbidopa	Terguride (dopamine agonist); domperidone in 4 subjects	Variable, 26–55 mg/h (from 5:00 a.m. until end of day's study)	55 mg/h	No side effects noted for L-dopa alone. For terguride plus levodopa, subjects had mild, transient asymptomatic orthostatic hypotension, headache, nausea, nervousness, drowsiness, light-headedness, and epigastric distress
Birkmayer and Hornykiewicz, 1962	Not given	Not given	None mentioned	None mentioned	50–150 mg	150 mg	No side effects mentioned
Birkmayer and Hornykiewicz, 1964	200	Not given	None mentioned	None mentioned	25 mg, once or twice a week, for up to 3 years	“Slow infusion”	Unclear whether L-dopa was administered without MAO inhibitors or nialamide. Nausea, vomiting and fainting were the major side effects which inversely correlated with the level of benefit
Birkmayer and Hornykiewicz, 1962	132	PD	None	MAO inhibitor (Ro-4/2637), caffeine, or euphyllin	50–150 mg infusions twice a week for 2 weeks	150 mg	L-dopa caused nausea and vomiting, if combined with MAO inhibitor. Caffeine or Euphyllin could reduce L-dopa side effects
Birkmayer and Mentasti, 1967	15	PD	Ro 4–4602 (benserazide)	None mentioned	50 mg	50 mg	No side effects mentioned. Decarboxylase inhibitor increased the benefit of L-dopa
Birkmayer, 1967	1	PD	None mentioned	None mentioned	50 mg	Not mentioned	No side effects mentioned
Birkmayer and Hornykiewicz, 1961	20	Parkinsonism (PD, postencephalitic parkinsonism, and vascular parkinsonism)	None mentioned	None mentioned	Up to 150 mg	“slow i.v.” Degkwitz et al., 1960	No side effects mentioned
Black et al., 2003	127	55 PD, 20 chronic tic disorders, 52 normal	Carbidopa	None mentioned	2.2 mg/kg	1.735 mg/kg /10 min	In healthy patients at high doses: nausea, vomiting, feeling uncomfortably hot, increased pulse rate. In PD patients at high doses: no side effects. In healthy patients at intermediate doses: nausea, vomiting. In PD patients at intermediate doses, some had dyskinesias but no nausea or vomiting. At low doses: there was some nausea in healthy patients
Black et al., 2010a,b	21	PD	Carbidopa	Tozadenant (SYN115)	0.6426 mg/kg	2.882 × 10^−5^ × (140–age) mg/kg/min	Carbidopa 200 mg was given by mouth at least an hour before the levodopa infusion began, using the method of Gordon et al. (2007) and a target plasma concentration of 600 ng/ml
Blanchet et al., 1999	8	PD (postmenopausal women with mild to moderate PD)	Carbidopa	Estradiol	29 ± 4 mg/10 min twice per day	33 mg/10 min	The threshold dose of levodopa necessary to provide definite antiparkinsonian efficacy was reduced significantly by 17[beta]-estradiol from 29 to 21 mg
Braun et al., 1987	7	Idiopathic PD	Carbidopa	SKF38393 (selective D-1 agonist) administered orally in double blind, placebo- controlled, crossover design	(10–80 mg/h) × 12 h	80 mg/h	No dyskinesias occurred with levodopa and simultaneous SKF38393 treatment. Dyskinesias at higher, supraoptimal doses. No side effects mentioned for L-Dopa alone: no orthostatic changes in blood pressure; patients remained asymptomatic throughout. Hematological parameters and blood chemistries remained within normal limits
Bredberg et al., 1990	5	PD (advanced)	Benserazide	None mentioned	Not given	1.5 mg/min	No side effects mentioned
Brod et al., 2012	12	PD	Carbidopa	None	2 mg/kg	1 mg/kg/h	Study compared low doses of carbidopa to higher doses. Side effects mostly related to parkinsonian symptoms associated with lower dose of IV levodopa than the patient's usual oral dose
Bronaugh et al., 1975	21	PD (15 idiopathic, 2 secondary to encephalitis lethargic, 2 associated with progressive supranuclear palsy)	None mentioned	None mentioned	calculated:30.8–56 μg (for 7 patients, and for 6 patients who were already on 3.0 g/day orally)	7.7–14 μg/4 h on top of an oral dose of 3.0 g/day	No side effects mentioned. Percent conjugation of L-dopa and metabolites given
Bruck et al., 1965	20	10 PD, 10 healthy	None mentioned	None mentioned	100 mg for PD, 50 mg for healthy individuals	50–100 mg/20–30 min	Nausea, lightheadedness, syncope, unpleasant sensation in head and abdomen, and increased blood pressure by 10–20 mmHg
Bruno and Brigida, 1965	18	Schizophrenia	None mentioned	Haloperidol	100–170 mg	2 mg/kg/5 min	No side effects mentioned for L-Dopa alone, only in combination with Haloperidol
Bruno and Bruno, 1966	40	Schizophrenia	None mentioned	20 received haloperidol, 20 received chlorpromazine	2 mg/kg	2 mg/kg/5 min	Neuroleptic-induced parkinsonism improved in both groups. Some improvement in antipsychotic-induced negative symptoms. Some patients developed nausea/vomiting, sweating, warmth/flushing, and dizziness (number not reported). No significant change in pulse or blood pressure
Camicioli et al., 2001	5	PD (idiopathic), functionally independent	Carbidopa	Methylphenidate (in one trial, compared to levodopa alone)	2 mg/kg	2 mg/kg/h	Apart from bothersome dyskinesias in one patient, patients did not report side effects or difficulties with treatments. No effect on heart rate or diastolic blood pressure, but L-Dopa led to a drop in systolic blood pressure. The drop in systolic blood pressure caused by L-Dopa was reduced when methylphenidate was administered alongside L-Dopa. No changes in mood, anxiety, arousal, or concentration before or after medications. Motor Unified Parkinson's Disease Response Scores were improved, as were tapping rates for both sides and both walking steps and rate
Chan et al., 2004	25	Idiopathic PD	Carbidopa	None mentioned	2.35 g/day (× 3 days)	1.6 mg/kg/h (× 2 h × 3 days)	No side effects mentioned
Chung et al., 2005	14	Idiopathic PD	Carbidopa	Paroxetine	2.0 mg/kg/day × 4 weeks	1.0 mg/kg/h	No side effects mentioned. No serious adverse effects
Chung et al., 2010	22	PD (15 with levodopa-induced dyskinesia)	Carbidopa	None mentioned	2–3 mg/kg	1.5 mg/kg/h	No side effects mentioned
Davis et al., 1991	10	Idiopathic PD	Carbidopa	None mentioned	None mentioned: just found “optimal dose rate” Total of 4 consecutive doses at the optimal rate were given, so highest total dose was 4.4 mg/day	1.1 mg/kg/10 min	Modest worsening of motor scores after levodopa stopped. Patients with unpredictable motor fluctuations have higher requirements for levodopa, both orally and intravenously, compared to those with simple wearing-off phenomena
Degkwitz et al., 1960	≥22	Psychiatric patients and normal controls	None	None mentioned	50–350 mg	Bolus (at least, ≤ 10 min)	No side effects mentioned
Durso et al., 1997	8	Idiopathic PD	Carbidopa	None mentioned	150 mg bolus	150 mg bolus stable isotope-labeled LD/12–15 min	No side effects mentioned
Durso et al., 2000	9	Idiopathic PD	Carbidopa	None mentioned	150 mg bolus	150 mg bolus ^13^C labeled L-DOPA/12–15 min	Average reduction in systolic blood pressure was 22 mmHg (14, 10–40). No prolonged cardiac arrhythmias were noted during infusion or subsequent 6-h monitoring
Fabbrini et al., 1987	28	Idiopathic PD	Carbidopa	None mentioned	1.5 mg/kg/h for ≥16 h	1.5 mg/kg/h	No side effects mentioned
Fabbrini et al., 1988	48	Idiopathic PD	Carbidopa	None mentioned	19.2 mg/kg	2.0 mg/kg/h	No side effects mentioned
Fasano et al., 1970a	66	PD	Benserazide 150 mg IV	Stimulant (“anfetamino- simile”)	Not stated	Not stated	The authors say, “no side effects were reported” with IV levodopa, whereas chronic oral levodopa dosing (without benserazide) produced side effects in 87% of patients (“psychic disturbances,” dyskinesias, nausea, vomiting, and orthostatic hypotension)
Fasano et al., 1970b	75	PD	None mentioned	None mentioned	Not stated	Not stated	No side effects mentioned
Fehling, 1966	25	PD	None	None mentioned	1.5 mg/kg	1.5 mg/kg over 13 min (6.9 mg/kg/h)	Levodopa did not differ from placebo in terms of clinical improvement. Levodopa caused a brief period of nausea in 9 patients and vomiting in 2 patients. Levodopa and placebo did not differ in their effects on blood pressure
Feigin et al., 2001	7	PD	None mentioned	None mentioned	Not given	100 mg/h (mean, 67.1 ± 25.6 mg/h)	No side effects mentioned
Feigin et al., 2002	7	PD	None mentioned	None mentioned	Varied	100 mg/h	No side effects mentioned
Feigin et al., 2003	7	PD	None mentioned	None mentioned	Varied	100 mg/h	Levodopa impaired aspects of sequence learning performance in non-demented PD patients; worsening in declarative score during motor sequence learning task suggests levodopa may have negative effects on aspects of cognitive processing linked to target retrieval. Levodopa also decreased activation of occipital association cortex during motor sequence learning
Friedhoff et al., 1963	11	Not given	None mentioned	None mentioned	Not given	2.5 mg/kg	No side effects mentioned
Gancher et al., 1987	20	PD (5 *de novo*, 4 stable, 11 fluctuating)	Carbidopa	None mentioned	1–4 mg/kg	0.5–0.8 mg/kg/h (lasting 2–5 h) for untreated PD. For treated PD, rate approximated usual oral LD dose	No side effects reported for IV L-dopa Infusions lasting 2–5 h. After oral levodopa, 2 of 5 *de novo* PD patients became nauseated (without emesis)
Gancher et al., 1988	33	PD (9 *de novo*, 7 stable responders, 17 fluctuating)	Carbidopa	None mentioned	0.8–3.0 mg/kg/h total (0.4 to 1.5 mg/kg/h × 2 h)	1.5 mg/kg/h	No side effects mentioned
Gerstenbrand and Pateisky, 1962	1	Parkinsonism due to post-encephalitis lethargica	None mentioned	None mentioned	200 mg	100 mg/20–40 min	Increased systolic blood pressure by 10 mmHg, mild mydriasis
Gerstenbrand and Prosenz, 1965	20	PD, postencephalitic parkinsonism and vascular parkinsonism	None mentioned	Isocarboxazid (MAO inhibitor)	50–75 mg/day for a few days, or with a few days interval between injections,. up to 6–8 injections total	Not given	L-dopa side effects included nausea, vomiting, blood pressure instability, and heat sensation. Subjects were pretreated with a MAO inhibitor (isocarboxazid) one tablet bid for 10–14 days
Gerstenbrand and Pateisky, 1963	30	Two with Huntington's Disease who had reserpine-induced parkinsonism; remaining subjects had postencephalitic parkinsonism, vascular or PD	None mentioned	MAO inhibitors	25–200 mg	100–200 mg/20–30 min (“infusion”), 25–75 mg/5 min (“injection”), 100 mg po	L-dopa side effects included: sensation of warmth in head, worsening of chorea in 2 Huntington's Disease subjects, nausea/vomiting, change in blood pressure beyond 20 mmHg, vertigo, syncope, unpleasant sensation in head and abdomen, and urge to urinate. Subjects underwent 14 days of pretreatment with MAO-inhibitors
Gillin et al., 1973	10	Mild to moderate depression (4 bipolar depression, 4 unipolar affective disorder, 1 cyclothymic personality, 1 borderline personality)	Carbidopa	None mentioned	25–50 mg	50 mg/2 min	Pre-REM infusions of L-dopa delayed the onset of REM sleep while infusion at REM onset shortened the length of the REM period. No detectable mood or side effects were noted except that three subjects had non-symptomatic reductions in blood pressure without change in pulse rate 5–25 min following the infusion
Goetz et al., 1998	5	PD w/daily visual hallucinations	Carbidopa	None mentioned	6 mg/kg (1.5 mg/kg/h × 4 h)	1.5 mg/kg/h	The authors tried to intentionally produce hallucinations in patients who had daily hallucinations with their usual treatment at home. IV doses were added to their oral medications. No patients developed hallucinations even though baseline dyskinesias persisted during the infusions
Goldstein et al., 1999	6	Healthy	None mentioned	None mentioned	99–118.8 μg/kg (0.33 μg/min/kg × 5–6 h)	0.33 μg/min/kg	No side effects mentioned. Authors suggest an enzymatic gut-blood barrier for detoxifying exogenous dopamine and delimiting autocrine/paracrine effects of endogenous dopamine generated in a “third catecholamine system”
Gordon et al., 2007	6	Healthy	Carbdiopa	None mentioned	Infusion over 90 min (total dose estimated at ~1100 mg)	Not given	No significant side effects; none of the side effects were above 1 (mild). Side effects included cold hands, mild irritability, headaches, nausea, stomach aches, but there were no significant differences between side effects reported by subjects on levodopa and those with placebo infusions
Gragnoli et al., 1977	25	8 healthy; 8 Diabetes Mellitus; 9 essential obesity	None mentioned	None mentioned	Not clear, possibly 1.5 mg/kg	1.5 mg/kg/10 min	None of the subjects suffered nausea or showed other signs of intolerance, or significant variations in blood pressure during the experiment. In diabetics and obese subjects, IV L-dopa causes a less marked human growth hormone increase than in control subjects, with diabetics having more of an increase than obese subjects
Gründig et al., 1969	14	9 PD, 5 normal	None mentioned	None mentioned	50 mg (control) to 100 mg	100 mg	No side effects mentioned
Hardie et al., 1984	20	Idiopathic PD	Carbidopa or benserazide	Apomorphine (dopaminergic agonist)	up to 1500 mg/day	80 mg/h	Dystonia and chorea. 4 patients experienced significant sleep benefit
Hardie et al., 1986	7	PD (on-off fluctuators)	PDI used but not specified	None mentioned	1280 mg (up to 16 h)	32–80 mg/h	No side effects mentioned
Hartvig et al., 1991	8	Healthy	1 subject given benserazide	None mentioned	5.5 mg or 11 mg	10 mg bolus	No side effects mentioned
Hashizume et al., 1987	6	Healthy	None mentioned	None mentioned	25 mg bolus	25 mg (bolus in 20 mL saline)	No nausea (except for one patient who was given oral levodopa); authors suggest that L-dopa undergoes decarboxylation and sulfation continuously even when administered intravenously
Henry et al., 1976	13	Depression, otherwise healthy	Carbidopa	None mentioned	50 mg (after a week's interval 6 pts got iv 50 mg DOPS or 100 mg L-DOPA without carbidopa)	50 mg/5 min	No nausea, vomiting, hypertension, or “other untoward side effects” The study was designed to “avoid such peripheral side effects by pretreating the patients with carbidopa.” IV levodopa was associated with reduced learning compared with chronic oral treatment and placebo infusions. No significant changes were found in heart rate/rhythm or blood pressure between levodopa and placebo
Hirano et al., 2008	11	PD	Carbidopa	None mentioned	Not given	0.56 mg/kg/h	No side effects mentioned
Hirschmann and Mayer, 1964a	10	PD	None mentioned	None mentioned	25–50 mg	50 mg	“No measurable, problematic side effects on the heart or circulation occurred with a slow IV injection of 25–50 mg”
Hirschmann and Mayer, 1964b	31	25 PD, 6 dystonia	None	MAO inhibitor	25–50 mg; 25 mg/day for 21 days; proceeded to year-long weekly and then monthly injections of unspecified amount	Not stated	No side effects mentioned
Horai et al., 2002	1	PD	Stopped	None mentioned	100 mg/h × 19 days	100 mg/h	Total dose ≈ 45,600mg. No side effects mentioned
Ingvarsson, 1965a	3	depression: long-standing, refractory (diagnosis unclear)	None mentioned	None mentioned	10–50 mg/day for weeks	50 mg/10 min	In one case, a sudden improvement in a concomitant asthmatic stridor was observed. “Depression” and “physical symptoms” improved in patients who were classified as depressed but may have had PD as well
Ingvarsson, 1965b	9	Not given	None mentioned	None mentioned	50 mg iv	50 mg	IV levodopa “abolishes asthmatic stridor”
Jaffe et al., 1987	6	PD	Carbidopa	None mentioned	≥2 h (at least 300 mg)	2.5 mg/min	One subject had mild dyskinesia. IV infusion of levodopa can affect the electroretinogram in patients with PD, indicating that the human retina is sensitive to changes in the systemic levels of levodopa and that this drug or its metabolite cross the blood-retinal barrier
Juncos et al., 1987	7	Idiopathic PD	Carbidopa	None mentioned	24 h/day × 6–13 days	~1.5 mg/kg /10 min (corrected for MW of L-Dopa instead of MW of L-Dopa methyl ester (LDME))	Motor fluctuations were markedly reduced with IV LDME. All patients noted an improvement in their condition during LDME treatment; reported benefits included improved sleep, attenuation of early morning akinesia or dystonia. There was no clinical or laboratory evidence of LDME toxicity
Juncos et al., 1990	12	PD	Carbidopa	None mentioned	1.6 mg/kg	7.1 ± 7.6 mg/h	Dyskinesia
Ko et al., 2013	14	PD			Not given	Not given, but see note	No side effects mentioned. Reportedly used same protocol as Mure et al. (2012) and Hirano et al. (2008)
Kobari et al., 1992	15	9 PD, 6 PSP (progressive supranuclear palsy)	None mentioned	None mentioned	1 mg/kg	2 mg/kg/h	No significant changes were noted in local cerebral blood flow after the administration of levodopa in patients with PSP
Kobari et al., 1995	34	16 idiopathic PD, 6 PSP (progressive supranuclear palsy), 5 olivopontocerebellar atrophy, 7 arteriosclerotic parkinsonism	Carbidopa	None mentioned	1 mg/kg	2 mg/kg/h	No significant changes in arterial blood pressure or heart rate. No side effects mentioned. Different patterns of regional cerebral blood flow response to levodopa in PD vs. PSP using xenon-enhanced CT
Lucas et al., 1975	33	18 healthy; 6 hypopituitarism; 9 chromophobe adenoma	None	None mentioned	100 mg	100 mg bolus (1.5 h after 25g arginine infusion)	No side effects mentioned
Maricle et al., 1995a	15	Idiopathic PD	Carbidopa	None mentioned	2 mg/kg	1 mg/kg/h	An elevation in mood ratings was seen for all 15 patients. (Mood ratings were an average of 40 before infusion, 60 during, and 42 after infusion). Mean anxiety decreased during the infusion (from 57 initially to 38 during infusion, and then increased to 62 after the infusion). Emotional fluctuations were seen in all patients, while only a third of the patients had a history of probable mood swings
Maricle et al., 1995b	8	idiopathic PD (and Fluctuating motor response)	Carbidopa	None mentioned	2 mg/kg daily × 3 days	1 mg/kg/h	Effect on mood and anxiety was dose responsive. Six of 8 patients had mood response (increase in mood score greater than 20%) during high dose infusion. Reduction of anxiety began shortly after onset of high-dose infusion. Peak effect on anxiety occurred 30 min after infusion had been stopped and was followed by precipitous increase in anxiety. Patients had little insight into discrepancy between their subjective reports and how they appeared to observers during their dyskinetic and agitated, but relatively euphoric state
Maricle et al., 1998	18	Idiopathic PD	None	Domperidone	2 mg/kg daily × 2 days	1 mg/kg/h	No significant side effects. Authors believe, “A significant mood response after a 2-day levodopa holiday supports the hypothesis that pharmacologic tolerance may be involved in this process and that sensitization may appear after a relatively brief period of abstinence form levodopa even in the first year of levodopa therapy”
Marion et al., 1986	3	PD	Benserazide	None mentioned	755–1750 mg/12 h	150 mg/10 min	No significant side effects mentioned. The patients did not experience any major discomfort or inconvenience during the course of the infusions and were pleased with their improved motor performance. Infusions were given for 6 h on day 1, and 12 h on day 2. One patient had mild dyskinesia. The number of on-off switches decreased and the duration of “on” periods increased in all three patients during the infusion periods compared to oral therapy. IV infusion of levodopa (with PDI) can give reproducible periods of constant mobility in selected patients for up to 5 consecutive days. One patient felt a feeling of “euphoria” after initial infusion. Another patient had a symptomatic fall of blood pressure from 140/80 mm Hg to 70/30 mm Hg when rate was at 99 mg/h of levodopa, so the infusion rate was decreased to 60 mg/h
Matussek et al., 1966	10	Depression and healthy subjects	None mentioned	None mentioned	25–50 mg, 50–100 mg	Not given	Headache, nausea
McGeer and Zeldowicz, 1964	10	PD	None mentioned	None mentioned	Not given	5 mg/min	Three patients who were given L-Dopa intravenously experienced nausea when infusion rate increased to 5 mg/min; but all pts tolerated 2 mg/min with no noticeable side effects, except for one patient who reported light-headedness immediately following the infusion
Metman et al., 1997	25	Advanced PD	Carbidopa	None mentioned	Max dose is 45–540 mg (15–180 mg/10 min × up to 3 doses)	180 mg/10 min	No side effects mentioned
Metman et al., 1999	4	PD	Carbidopa	None mentioned	413–483 mg (64 ± 5 mg/h × 7 h)	69 mg/h	No side effects mentioned
Metzel, 1965	61	PD	None mentioned	MAO inhibitor	Not given	Not given	No side effects mentioned. In some cases dopa was combined with a MAO-inhibitor
Moorthy et al., 1972	8	Organic heart disease undergoing routine catheterization	None mentioned	None mentioned	100–200 mg (avg. 144 mg)	200 mg/10 min	Nausea (5 pts), accompanied by vomiting (in 2 pts). The nausea was severe at 10–15 min after the start of the L-dopa infusion. Serious arrhythmias were not seen. Two patients had ventricular premature contractions. The effects on the cardiovascular system observed were slight. blood pressure showed a tendency to fall in some patients during the initial 5 min after injection and to rise later to values higher than the control values. No serious complications were seen. The authors' observations seem to indicate that treatment with L-dopa is not particularly dangerous in patients with organic heart disease
Mouradian et al., 1987a	23	PD	Carbidopa	None mentioned	Up to 11 days, 24 h/day	1.8 mg/kg/h	Maximum rate provided in Juncos et al. (1990), who also give number of subjects as 28. No side effects
Mouradian et al., 1987b	4	Idiopathic PD	Carbidopa	None mentioned	Optimal dose infusion (not quantified)	Optimal dose rate lasting at least 16 h	No side effects mentioned; no cardiovascular complications. There was no discernible alteration in the motor response to intravenous levodopa at any time during the period of physical activity
Mouradian et al., 1988	29	Idiopathic PD	Carbidopa	None mentioned	200 mg	200 mg/10 min	No side effects mentioned
Mouradian et al., 1990	12	PD	Carbidopa	None mentioned	1.0 ± 0.1 mg/kg/h × up to 12 days	variable; apparently up to 200 mg/10 min as a loading dose	Minimal dyskinesias, with 1.0 mg/kg/h as the dyskinesia threshold dose
Mure et al., 2012	8	PD	None mentioned	None mentioned	1.13 ± 0.41 mg/kg/h (duration not reported)	Not given	Doses titrated to achieve maximal Unified Parkinson's Disease Rating Score response without causing dyskinesia. No significant changes in regional cerebral blood flow
Nardini et al., 1970	17	PD	None mentioned	None mentioned	25 mg	25 mg “slow infusion,” 1.5 - 3 mg/kg/h	Asthenia, insomnia, anxiety, headache, increased “tensori,” restlessness, disorientation and confusion. No side effects in arterial pressure, digestive problems, liver or renal function
Nisijima et al., 1997	3	Neuroleptic malignant syndrome (NMS)	None mentioned	Two patients infused with dantrolene	50–100 mg/day	Not given	No side effects mentioned. Symptoms of NMS decreased dramatically. Authors write, “Levodopa, particularly in injectable form, should be more positively used for pharmacotherapy in patients with NMS”
Nutt et al., 1984	9	Idiopathic PD	Carbidopa	None mentioned	Total between 2200–7200 mg, (infusions were continued for 20–36 h)	110 mg/h with carbidopa, 200 mg/h without carbidopa	Severe dyskinesia in one patient. The patients moved around the ward and exercised freely due to IV L-dopa. Eating a high-protein meal during levodopa infusion is associated with a decline in the clinical response to the infused levodopa without any alteration in the plasma concentration
Nutt et al., 1985	9	Idiopathic PD	Carbidopa	None mentioned	Max 1250 mg	2.12 mg/kg/h	Mild dyskinesia
Nutt et al., 1988	8	PD (with fluctuating response)	Carbidopa	None mentioned	0.28–2.54 mg/kg	1.27 mg/kg/h	Post-improvement worsening. Some mild dyskinesia
Nutt et al., 1992	27	PD	Carbidopa	None mentioned	0, 0.4, 0.8, 1.6, 2.4 mg/kg/h × 2 h	2.4 mg/kg/h	Mild dyskinesia
Nutt et al., 1993	19	PD	Carbidopa	None mentioned	33.3 mg/kg/ 21 h	1.6 mg/kg/h	Short infusions were well-tolerated, long infusions less so. Two subjects had dyskinesia during long infusion and two others suffered from confusion, although short infusions were well-tolerated by all subjects
Nutt et al., 1994	17	Idiopathic PD	Carbidopa	None mentioned	2 h (average 1.96 mg/kg, max 3.2 mg/kg)	Max: 1.6 mg/kg/h, mean: 0.98 mg/kg/h	2 patients developed nausea and one experienced lightheadedness (only during post-holiday levodopa infusions). In general, 2-h levodopa infusions were “well-tolerated,” with no medical complications during the levodopa holiday
Nutt et al., 1995	16	Idiopathic PD	Carbidopa	None mentioned	2 mg/kg	mean 0.98 mg/kg/h	Some nausea and lightheadedness
Nutt et al., 1997a	11	Idiopathic PD (and fluctuating response)	Carbidopa	None mentioned	2 mg/kg	1.51 mg/kg/h	Mild dyskinesia
Nutt et al., 1997b	18	PD	Carbidopa	Domperidone	4 mg/kg total (2 mg/kg daily × 2 days)	1 mg/kg/h	Levodopa therapy was able to restore tapping speed almost to normal
Nutt et al., 2001	12	Idiopathic PD	Carbidopa	None mentioned	2 or 3 mg/kg	1 or 1.5 mg/kg/h	No side effects mentioned. Mood, anxiety, and blood pressure were measured at 30-min intervals for 7 h total, and there was no mention of any effects of levodopa on anxiety or blood pressure
Nutt et al., 2002	18	Idiopathic PD	Carbidopa	Domperidone	4 mg/kg total (1 mg/kg/h × 4 h)	1 mg/kg/h	The same dose of L-Dopa produced progressively more severe dyskinesia with long-term L-dopa therapy but did not increase the duration of dyskinesia in patients. However, increasing the dose of L-dopa in subjects with dyskinesia does not increase the severity of dyskinesia but does increase the duration of dyskinesia
Nutt and Nygaard, 2001	4	All 4 had DRD (dopa-responsive dystonia); 2 had PD in addition to DRD	Carbidopa	None mentioned	2 mg/kg daily × 2 days	1 mg/kg/h	No side effects mentioned. “In one subject, two doses of levodopa and a night's sleep abolished her dystonia and restored normal tapping rate”
Nutt and Woodward, 1986	23	Idiopathic PD (and fluctuating response)	Carbidopa	None mentioned	3.0–13.2 mg/kg (0.5–2.2 mg/kg/h × 6 h)	2.2 mg/kg/h	2 patients exhibited a brief burst of mobility and dyskinesia lasting minutes. Generally, with the onset of mobility, the patients had a brief burst of tremor, or tremor mixed with dyskinesia, and then became mildly dyskinetic
Ogawa et al., 2012	1	PD	None mentioned	Dai-kenchu-tou (5-HT3 receptor agonist)	Not mentioned	75 mg/kg daily boluses, duration not reported	IV levodopa was used as a treatment for neuroleptic malignant syndrome
Oishi et al., 1996	20	Parkinsonism (PD, vascular parkinsonism)	None mentioned	None mentioned	50 mg	50 mg bolus	No side effects mentioned
Pare and Sandler, 1959	3	Depression candidates for ECT who were responsive to Iproniazid	None mentioned	Iproniazid	12.5–137.5 mg (25 mg–275 mg racemic)	275 mg bolus of DL-DOPA	No side effects mentioned. DL-DOPA was used
Pazzagli and Amaducci, 1966	11	PD	None mentioned	None mentioned	60 mg, 90 mg, or 120 mg	Not given	Hypotension, nausea, vomiting, somnolence, and mild sedation accompanied by feeling euphoric
Peppe et al., 1991	5	PD	Carbidopa	Domperidone	770 mg/day × 5 days, (given 110 mg/kg/h × 7 h)	110 mg/h (mean 70 mg/h)	No side effects mentioned
Poewe, 1993	Not given	Not given	None mentioned	None mentioned	Not given	not given	No side effects mentioned. From a table entry in a review article
Pullman et al., 1988	10	5 PD and 5 healthy	Carbidopa	None mentioned	Not given	Varied rates from high, middle, and low (actual dose not specified)	No side effects mentioned
Puritz et al., 1983	13	6 healthy; 7 progressive autonomic failure and multiple system atrophy (MSA)	None mentioned	None mentioned	99.875 mg	1.175 mg/min	No subjects experienced adverse effects during the infusion although one vomited after discontinuation of L-dopa. For one dosage and rate: change in AVP (plasma arginine vasopressin), blood pressure and heart rate are given. No significant effects of L-Dopa on mean blood pressure in normal subjects, but lowered blood pressure of MSA patients. No effect heart rate or AVP levels in basal state. Author suggests “L-Dopa should not be prescribed for patients with MSA”
Quinn et al., 1982	3	PD	Benserazide	None mentioned	Not given (only that treatment was given for about 8 h at unspecified rate)	Not given	No side effects mentioned. The patients with severe on-off fluctuations had dramatic benefit. Authors write, “Intravenous levodopa infusion obviously overcomes many of the problems of intermittent oral treatment”
Quinn et al., 1984	10	PD	Carbidopa or benserazide	None mentioned	Variable; highest total dose appears to be 187 mg/h × 8.8 h × 12 doses	150 mg bolus in ≥2 subjects; all subjects received 100–200 mg over 10 min, then up to 187 mg/h (mean 125 mg/h)	Pulse and blood pressure fell, but to the same degree as with oral levodopa; “slight and transient” postural faintness (orthostasis); coldness of the limbs; nausea and vomiting; dyskinesias. No patient complained of palpitations during the infusions, and no arrhythmias were detected. Authors assert, “Continuous intravenous infusion of levodopa turns out to be the most effective way of abolishing the off state during a substantial period of the day”
Rinne and Sonninen, 1968	36	Idiopathic PD (24) and post-encephalitic PD (12)	None	None mentioned	1.5 mg/kg	1.5 mg/kg/10 min	Pulse and blood pressure changes were comparable between levodopa and placebo. Common adverse effects included nausea (47%), vomiting (31%), vertigo (19%), headache (33%), sweating (44%), and anxiety (22%); frequency of adverse effects not reported with placebo
Roberts et al., 1995	8	Normal	Carbidopa	None mentioned	50 mg	50 mg/5 min	None mentioned
Robertson et al., 1989	28	12 healthy elderly and 16 healthy young subjects	Both with and without carbidopa	None mentioned	50 mg bolus	50 mg bolus/5 min	None mentioned
Rodriguez et al., 1994	14	Asymmetric PD	Carbidopa	Domperidone. Apomorphine given subcutaneously	960 mg to 2200 mg (60–100 mg/h × 16–22 h)	250 mg/h (for short infusion). Up to 100 mg/h (for long infusion)	None mentioned
Rosin et al., 1979	1	Idiopathic PD and carcinoma of the rectum	Carbidopa	None mentioned	4320 mg highest total dose for a day (given between 1200 and 4320 mg/day for 7 days)	180 mg/h	No side effects or adverse effects: no “undue” abdominal distention, nausea, vomiting, cardiac arrhythmia, or hypotension
Ruggieri et al., 1988	20	Idiopathic PD	Carbidopa	Domperidone	(360–1200 mg/day) × 3 days	1200 mg/day x 3 days	The patients were given constant IV L-dopa infusion for 12 h × 3 days. Mild somnolence, nausea, and occasional vomiting were the only side effects reported. There was an increase in blood pressure (probably due to domperidone). Maximum optimal drug rate ranged from 30–104 mg/h with mean 53.5 mg/h
Sage and Mark, 1991	1	PD	Carbidopa	None mentioned	240 mg/day (during nighttime)	30 mg/h	No side effects mentioned. Oral carbidopa/levodopa was given during the daytime while IV levodopa was administered at night. Nighttime infusions produced immediate benefit of a good night's sleep, and nighttime levodopa infusions also reduced patient's daytime motor fluctuations. Authors suggest the levodopa infusion rate required to produce the best results was between 40 and 45 mg/h
Sasahara et al., 1980b	5	PD	None mentioned	None mentioned	50 mg	50 mg/20 min	No side effects mentioned
Schuh and Bennett, 1993	6	Advanced idiopathic PD	Carbidopa	None mentioned	57.6 mg/kg, (given 24 h/day × 3–8 days)	2.4 mg/kg/h	L-Dopa induced dyskinesia, but only occurs because of the progression of PD. No other side effects mentioned
Shinoda et al., 2013	1	PD	None mentioned	None mentioned	75 mg	50 mg bolus	Patient developed neuroleptic malignant syndrome (NMS) due to underdosing of IV levodopa as a result of dilution in extracorporeal circulation during open heart surgery
Shoulson et al., 1975	5	PD	Carbidopa	None mentioned	Not given (duration of 3 h at unspecified rate)	Not given	No side effects mentioned. No significant changes in pulse rate or blood pressure occurred
Siddiqi et al., 2015	29	Tourette syndrome and healthy controls	Carbidopa	None	0.6426 mg/kg	2.882 × 10^−5^ × (140–age) mg/kg/min	No significant difference in pulse, blood pressure, or orthostatic change between IV levodopa and placebo when co-administered with carbidopa
Skalabrin et al., 1998	9	Advanced PD	Carbidopa	None mentioned	Not given	2.6–3.0 mg/kg/h	Doses escalated until a maximum of 3.0 mg/kg/h infusion rate was achieved, OR the subject experienced maximum dyskinesia, or developed nausea or hypotension
Sohn et al., 1994	42	PD	Carbidopa	None mentioned	36–150 mg	150 mg/10 min	No side effects mentioned
Souvatzoglou et al., 1973	25	Healthy	None mentioned	None mentioned	1 mg, 5 mg, 12.5 mg, 25 mg, or 100 mg	5 mg/ml L-dopa infused, blood samples drawn at 10 min intervals over 3–4 h. (therefore the lowest max rate possible was 5 mg/ml/min)	2 cases at 100 mg of mild nausea lasting 5–10 min. In no instance were any cardiac effects observed. Serum growth hormone is stimulated by 25 mg IV L-dopa
Stocchi et al., 1986	18	Idiopathic PD	Carbidopa	None mentioned	1080–3750 mg total (360–1250 mg/day for 3 days)	1250 mg/day × 3 days	No side-effects, except for a mild somnolence during the first day, were recorded. Blood pressure, cardiac electric morphology, and rhythm did not change significantly during the study. Authors argue IV infusion could be a precious form of rating the real single individual's L-dopa needs. They write, “L-dopa infusion remains a good technique in the overall evaluation of the parkinsonian patient and indispensable in particular situations like post-operative recovery and intensive care”
Stocchi et al., 1992	9	PD	Carbidopa	None mentioned	100 up to ≥600 mg/days	200 mg boluses or 400 mg/h. For 3 subjects: “optimum rate” for 12 h × 3 days	No side effects mentioned. Blood pressure and pulse were assessed every 15 min, and no mention was made of any changes to either blood pressure or pulse
Sunami et al., 2000	1	Akathisia	None mentioned	None mentioned	25 mg/day × 8 days, followed by lower infusions	25 mg/day	No side effects mentioned. Authors believe IV levodopa treatment “would be useful in reducing the persistent neurotoxicity (lethargy, hypersomnia, depression, agitation, akathisia, and confusion) associated with interferon-alpha”
Takeuchi et al., 1993	8	Healthy	None mentioned	None mentioned	50 mg	50 mg for >10 min	Study of mechanisms of orthostatic hypotension in L-dopa treated PD. At rest, the systolic blood pressure was significantly lowered by L-dopa administration, but diastolic blood pressure, heart rate, and calf blood flow were not significantly altered by L-dopa administration. Spontaneous muscle sympathetic nerve activity was significantly higher than that before administration. Results support hypothesis that L-dopa and/or its metabolites act on peripheral blood vessels at sympathetic nerve terminal, thereby inducing orthostatic hypotension
Takubo et al., 2003	32	Malignant syndrome (MS)	Some subjects given unspecified PDI	None mentioned	2440 mg/day (for patient before study began)	Not given	No side effects mentioned. Suggests the following dosages of IV levodopa in the treatment of malignant syndrome: 300–600 mg/24 h or 100–200 mg/3 h three times a day
Tedroff et al., 1990	6	PD and healthy	Benserazide	None mentioned	0.9 mg	0.9 mg bolus	No side effects mentioned
Tedroff et al., 1992	8	Idiopathic PD	Benserazide	None mentioned	200 mg	200 mg/6 min	No side effects mentioned; brain uptake of [β-^11^C]-L-DOPA was inversely correlated to the sum of large neutral amino acids in plasma
Tedroff et al., 1996	10	PD	Carbidopa	None mentioned	3 mg/kg	0.5 mg/kg/min bolus for 5 min	Before the study, one patient was excluded due to levodopa-induced nausea. Authors write, “levodopa is still the most effective symptomatic treatment for PD, and compared with the various dopamine agonists available, is well-tolerated by most patients. The finding that the capacity for levodopa to produce increased synaptic dopamine levels is most profound in the more denervated regions of the striatum means that levodopa is acting preferentially at the site of dopaminergic denervation”
Torstenson et al., 1997	10	Idiopathic PD	Carbidopa	None mentioned	5 mg/kg (2 mg/kg + 2 mg/kg/h × 1.5 h)	0.5 mg/kg/min over 4 min as bolus; then 2 mg/kg/h	No side effects mentioned
Tzavellas and Umbach, 1967	125	PD	None mentioned	Propylhexadrine (amphetamine)	Not given	Not given	No side effects mentioned. Subjects received a combination of L-dopa and propyl-hexedrin (MAO inhibitor)
Umbach, 1966	Not given	Not given	None mentioned	Amphetamine	Not given	Not given	No side effects for L-dopa alone. Reported side effects are caused by combination treatment with amphetamines. L-dopa and amphetamine treatment of akinetic Parkinsonism patients with and without stereotaxic surgery. It is not clear how many were treated only with L-Dopa
Umbach and Baumann, 1964	35	30 PD, 5 controls	None mentioned	None mentioned	100 mg in 13 patients and 100 mg in 17 patients	Not given	Patients after stereotaxic surgery. Specific L-dopa side effects are not mentioned, but it is said that higher doses caused more severe side effects
Umbach and Tzavellas, 1965	30	PD	None mentioned	Propylhexadrine (amphetamine)	50 mg	Not given	L-Dopa alone caused drop in blood pressure
Verhagen Metman et al., 1998a	6	Idiopathic PD	Carbidopa	Dextromethorphan	up to 65 ± 14 mg	Not given	No side effects mentioned. Brief IV infusions (10 min each, 4 h for a total of 9–12 infusions)
Verhagen Metman et al., 1998b	14	PD	Carbidopa	None mentioned	≥150 mg	150 mg/10 min	No side effects mentioned
Voller, 1968	180	PD	None mentioned	In unspecified number of patients, MAO inhibitors (isocarboxazid 10 mg TID or nialamide 25 mg BID)	25 mg twice per week	Not given	Increase of PR interval (on electrocardiogram), tachycardia, sweating, nausea. All these were mild and transient so that no experiment was interrupted
Worth et al., 1988	6	Healthy	None mentioned	None mentioned	840 μg/kg	7 μg/kg/min	Mean plasma renin activity fell by 50%; significant increase in urinary sodium excretion and effective renal plasma flow; mean diastolic blood pressure fell with no reflex tachycardia. Mean diastolic pressure fell on infusion of L-dopa. Trends toward fall in mean systolic pressure and rise in mean pulse rate on infusion of L-dopa, but these were not significantly different from changes occurring on saline infusion
Zsigmond et al., 2012	10	PD	None	None	281.25 mg	375 mg/h for 45 min	No side effects mentioned. In 2 patients who had previously discontinued oral levodopa/carbidopa due to nausea, high doses of IV levodopa were well-tolerated and relieved symptoms
Total references 142	Total 2760						

*Summary of published studies reporting IV levodopa use in humans, 1959 to early 2015*.

Concomitantly administered peripheral decarboxylase inhibitors included carbidopa and benserazide. PDIs affected clearance and volume of distribution (as mentioned above), minimized gastrointestinal symptoms, and allowed subjects to be given lower doses of levodopa. Other concomitant drugs are listed, to help explain any side effects that might be caused by concomitant drug administration or an interaction with levodopa rather than by levodopa alone. These include adenosine receptor antagonists (istradefylline, tozadenant [SYN115], aminophylline, caffeine), stimulants (amphetamines, methylphenidate), dopamine receptor agonists (apomorphine, terguride, SKF38393), monoamine oxidase (MAO) inhibitors, dextromethorphan, estradiol, paroxetine, and dantrolene.

A variety of neurological, psychiatric, cardiovascular, and other physiological effects of levodopa were monitored (see Table 1). There were no reported deaths. There were no instances of psychosis, even when attempting to elicit it in susceptible subjects (Goetz et al., 1998). There were also no life-threatening events (serious adverse effects) following IV levodopa administration at high doses, regardless of whether a PDI was co-administered. With co-administration of a PDI, the dosage range causing side effects (mainly nausea and asymptomatic hypotension) was 45–150 mg as a single bolus or infusions of 0.5–2.0 mg/kg/h. Without a co-administered PDI, side effects were reported with a bolus of 60–200 mg or an infusion of 1.5–3.0 mg/kg/hr. Side effects were more likely with higher doses, but other factors such as age, sex, disease severity, and prior treatment also played a role in side effects of levodopa.

Other than these side effects found at high doses, several milder or less frequent side effects were reported. These primarily included mild autonomic changes (orthostasis and tachycardia), psychiatric changes (sedation, anxiety, insomnia, and improvement in mood), and neurologic effects (improvements in tics, REM sleep changes, subjective weakness, headaches, and increased dyskinesias). Various other effects were noted in isolated reports (listed in Table 3). It is important to note that both side effects and efficacy depended strongly on subject factors including gender, age, past treatment, and disease state. Also, dsykinesia was mentioned as a side effect only in patients with PD, and most often in those with a long history of previous levodopa treatment.

Motor benefits of levodopa in PD have been demonstrated conclusively. Additional reported benefits of IV levodopa treatment in PD included improved sleep (Hardie et al., 1984) and attenuation of early morning akinesia or dystonia (Juncos et al., 1987). In other patient groups, benefits of IV levodopa included improvement of the comatose state in hepatic encephalopathy (Abramsky and Goldschmidt, 1974) and improvement in depressive and somatoform symptoms (Ingvarsson, 1965a). One report found it more effective than dantrolene for treating neuroleptic malignant syndrome (Nisijima et al., 1997). More recently, IV levodopa treatment was found to alleviate the neuropsychiatric adverse effects associated with interferon-alpha, namely lethargy, hypersomnia, depression, agitation, akathisia, and confusion (Sunami et al., 2000).

## Discussion

The existing literature strongly supports the safety of IV levodopa, which has been used in humans for more than half a century (Pare and Sandler, 1959). IV levodopa has been administered to over 2700 human subjects. Despite infusion rates as high as 5.0 mg/kg/h and boluses as large as 200 mg, there are no recorded instances of death or of other serious adverse effects of IV levodopa, nor have there been documented cases of other serious side effects, such as psychosis, that might limit its use in humans. Milder side effects, the most significant of which are nausea and vomiting, were most prominent with rapid infusions in the range of 1–2 mg/kg or 100–200 mg over less than 15 min (Bruno and Bruno, 1966; Fehling, 1966; Rinne and Sonninen, 1968; Moorthy et al., 1972; Quinn et al., 1984; Black et al., 2003).

These conclusions are supported by safety data from other species. The Registry of Toxic Effects of Chemical Substances reports the lowest published toxic dose of levodopa in any non-human species as 2.5 mg/kg, referring to a subtle behavioral effect on a learning measure in a mouse (NIOSH and Biovia, 2015)^1^. The lowest IV levodopa dose that was lethal to half of subjects (LD50) was “>100 mg/kg” in rats. In mice, the LD50 ranges from 450 mg/kg (administered intravenously) to 4449 mg/kg (administered subcutaneously). Typical human doses are in the range of only 1 mg/kg; thus, human studies with IV levodopa administer doses substantially lower than those dangerous to nonhuman mammals.

In summary, IV levodopa has similar efficacy and side effects as oral levodopa (Connolly and Lang, 2014) and dopamine agonists (Bonuccelli and Ceravolo, 2008). These include gastrointestinal (nausea, vomiting, and abdominal discomfort) and neuropsychiatric effects (sedation, dyskinesias). Nausea and orthostatic hypotension, side effects of both IV and oral levodopa, are largely blocked by PDIs and are less common in patients accustomed to dopamimetic treatment. The other side effects are infrequent and neither serious nor life-threatening (Connolly and Lang, 2014). When given with adequate PDI pretreatment, IV levodopa has minimal if any cardiovascular effects (Siddiqi et al., 2015).

The safety of IV levodopa is important for patients but also for regulatory review. Changing the route of administration of any drug in a study traditionally necessitates submitting an IND application if changing the route of administration “significantly increases the risks … associated with the use of the drug product” [§21 CFR 312.2(b)(iii)]. The data from our review of the literature suggest that IV administration of levodopa does not significantly increase the associated risks of levodopa in comparison to oral administration. In summary, studies conducted throughout the past half century support the safety of IV levodopa administration in human patients.

## Author contributions

Literature search: NA, SS, CG, KB. Writing: SS, CG, JP, KB. Statistics: NA, KB. Translation from German: MK. All authors approved the final manuscript.

### Conflict of interest statement

The authors declare that the research was conducted in the absence of any commercial or financial relationships that could be construed as a potential conflict of interest. Kevin J. Black is Sponsor-Investigator for an Investigational New Drug application for intravenous levodopa (U.S. FDA).

## Footnotes

^1^RTECS reported the lowest toxic dose as “100 μg/kg,” but the dose in the cited reference was actually 100 μg/g = 100 mg/kg (Takahara et al., 1980).
